# Screening for anxiety and its determinants among secondary school students during the COVID-19 era: a snapshot from Qatar in 2021

**DOI:** 10.1007/s44192-022-00014-1

**Published:** 2022-05-30

**Authors:** Alia Albinali, Sarah Naja, Noora Al Kaabi, Nagah Slim

**Affiliations:** 1 Hamad Medical Corporation, Doha, Qatar; 2 Primary Health Care Corporation, Doha, Qatar; 3 Community Medicine Residency Program, Department of Medical Education, Hamad Medical Corporation, P.O. Box 3050, Doha, Qatar

**Keywords:** Anxiety, Screening, GAD-7, COVID-19 strategies

## Abstract

**Background:**

Anxiety among adolescents may lead to disability and has a tremendous impact on one’s quality of life. The alarming COVID-19 pandemic is expected to increase the anxiety level of adolescents especially with enforced governmental management strategies. This study will assess anxiety symptoms among secondary school students during the COVID-19 pandemic in Qatar.

**Methods:**

We conducted an analytical cross-sectional study among adolescents attending independent secondary schools in Qatar. First, potential participants were invited through Microsoft teams. Next, a total of 750 participants were assessed through the Generalized Anxiety Disorder-7 (GAD-7) tool. We then conducted descriptive analyses and the Chi-square test to examine significant determinants of anxiety, which was followed by logistic regression analysis. In the end, the scale was tested for its internal consistency using Cronbach’s alpha.

**Results:**

Anxiety symptoms were seen in 37.2% of the participants. Female gender, previous history of mental illness, comorbidities, permissive parenting style, and spending more than 12 h per day on the internet were significant determinants of anxiety. Furthermore, a previous history of mental illness, low perceived social support, isolation, and social distancing predicted anxiety.

**Conclusions:**

Anxiety is common among secondary school students in Qatar, and preventive interventions must target the determinants, especially during a pandemic.

## Background

Adolescents are a vulnerable group, as it is a time of difficult transition from an emotional and developmental prospective [1]. Before the COVID-19 pandemic, approximately 10–20% of children and adolescents worldwide were affected by mental health problems [2]. Notably, the World Health Organization declared mental health among adolescents as a public health problem, accounting for 16% of the global burden of disease and injury. Specifically, anxiety disorder is the ninth leading global cause of disease and disability for adolescents [3]. A systematic review conducted among the six Gulf Cooperation Council (GCC) countries (Bahrain, Kuwait, Oman, Qatar, Saudi Arabia, and the United Arab Emirates) revealed that the pooled prevalence of anxiety ranged between 17.27% and 57.04% [4].

The coronavirus has been widely publicized as a “killer disease,” leading to higher anxiety among the public. At the same time, governments have adopted strict measures, including travel bans, forbidding large gatherings, suspension of public transportation, closure of schools and universities, and social distancing to break the chain of infection and control the pandemic [5].

These restrictions and the uncertainty of the disease have impacted the daily lives of people of all ages and can significantly affect mental well-being [6, 7]. Specifically, adolescents are becoming more afraid, angry, anxious, and stressed. Unfortunately, if anxiety is left undiagnosed and untreated, it can negatively impact adolescents’ development, social life, and future careers [8].

Globally, few studies have investigated the effect of the COVID-19 pandemic on adolescents’ anxiety levels, and these have been mainly in response to public health emergencies. Specifically, in China, two out of ten adolescents experienced anxiety during the pandemic [9]. Furthermore, in Italy, anxiety symptoms were self-reported higher during the pandemic, although none of the participants were affected by the COVID-19 infection [10].

Poor mental health is a growing problem for adolescents, leading the Qatar government to prioritize adolescents' mental health in their national health strategy. Demographically, the youth population represents around 25% of the people in Qatar [11]. Under the influence of the COVID-19 pandemic, the Ministry of Public Health adapted strategies to tackle the pandemic. Adolescents were separated from their friends, quarantined, wore masks, and received online education [12].

### Purpose

The current study aims to determine the anxiety level in a sample of healthy older adolescents in a secondary school setting in Qatar. It was hypothesized that because of the effects of the COVID-19 pandemic, the sample would show a high level of anxiety.

## Methods

### Study design and setting

We used a cross-sectional research method. The adolescents were selected from independent secondary schools, as they represent healthy adolescents living in the community. Independent secondary schools are distributed throughout Qatar; the total is 68, divided into 34 schools for boys and 33 for girls, since the population of our focus is school-aged adolescents. Independent secondary schools are free public government-funded schools for adolescents in Qatar. The total number of students in independent secondary schools is 28,609. Of this total, 6,933 are male Qatari students and 6,786 are non-Qatari male students. A total of 8,043 students are Qatari females and 6,847 of the females in the independent secondary schools are non-Qatari.

### Study population and sampling technique

The study population included students ages 14–19 who were enrolled in independent secondary schools in grades 10–12 in Qatar for the 2020–2021 academic year. There were no restrictions on nationality and gender. Those on leave from school were excluded. The sampling technique was non-probability sampling.

### Sample size and enrollment of participants

The sample size was calculated using the following equation [13]:

$${\text{n }} = \, \left[ {{\text{Np}}\left( {{1} - {\text{p}}} \right)} \right] \, / \, [{\text{d2}}/{\text{Z21}} - \alpha /{\text{2 x }}\left( {{\text{N}} - {1}} \right) \, + {\text{ p x }}\left( {{1} - {\text{p}}} \right)]$$

where:

n: Target population (a total of 28,609 registered 14–19 years old children in independent secondary schools) according to the Ministry of Education and Higher Education (MOEHE) main statistics for the academic year 2019–2020.

n: Sample population (minimum number of the required sample size) [14]

P: Probability or prevalence of the psychological impact of COVID-19 pandemic among independent secondary school students in Qatar 2020 will be 50% to yield maximum sample size.

d: Acceptable error rate or absolute precision on either side of the proportion: 5% = (0.05).

Z: Statistics for an error of 0.05 corresponding to a 95% confidence level = (1.96).

### Data collection

Due to social distancing and less human contact, data collection was conducted online. Self-administered online data collection tools were sent to all independent secondary schools in Qatar via Microsoft teams. The representative social worker from each school forwarded the online questionnaire to secondary school students. All eligible students were enrolled in the study after an online consent was sent to parents, and an assent form was sent to students. When the minimum sample size was achieved, the response tab was closed.

### Variables and measures

#### Anxiety (dependent variables)

Anxiety is a feeling of worry or fear that can be mild or severe. Using the General Anxiety Disorder-7 (GAD-7), participants were asked how often they were bothered by each symptom during the last two weeks. The tool consists of seven questions, and the total score range from zero to twenty-one [15]. Based on the severity of the reported symptoms, the participants showed minimal anxiety (total score 0–4), mild anxiety (total score 5–9), moderate anxiety (total score 10–14), and severe anxiety (total score ≥ 15). Therefore, the higher the score, the higher the anxiety symptoms [16].

The scale has been used in many studies to assess anxiety symptoms in adolescents. The questionnaire is available in English. However, the Saudi Arabic version of GAD-7 has been validated and was, therefore, employed to assess Qatari adolescents’ severity of anxiety [17].

The psychometric properties of the General Anxiety Disorder-7 (GAD-7) were tested across many countries and showed strong, pooled sensitivity and specificity around 80% at a cut-off point equal to ten [18]. Furthermore, the same cut-off point was utilized to distinguish anxious from non-anxious adolescents in Qatar [19].

#### Determinants (independent variables)

##### Sociodemographic, behavioral and COVID-19 related strategies

After being tested for face and content validity by field experts, Arabic versions of questionnaires were utilized to measure independent variables. Using Lawshe's method, the experts judged the content validity of each item for its relevance and importance on a 3-point rating scale: (1) not necessary, (2) useful but not essential, and (3) essential. The universal agreement between the three raters was 90%. Finally, a sample of ten questionnaires was administered to adolescents to ensure their accuracy, and these were excluded from analysis.

The structured, comprehensive, multi-component questionnaire included questions on adolescents’ sociodemographic characteristics (age, gender, nationality), educational background (academic performance, grade level), perceived educational strategy (remote, blended), health-related factors (acute, chronic illness, previous history of anxiety, family history of mental illness, medications), social factors (parenting style), behavioral factors (internet use), COVID-19 related information, and practice (history of infection, mask wearing, social distancing, quarantine, hand hygiene).

##### Perceived social support

The perceived social support includes all individuals who were a part of the adolescents’ social network (family, friends, and teachers). This is defined by the belief in the availability of support regardless of whether the support is available or not. It refers to the experience of being valued, respected, cared about, and loved by others who are present in one's life from different sources, such as family, friends, teachers, community, and any social groups to which one is affiliated [20].

The Multidimensional Scale of Perceived Social Support (MSPSS) was used to assess perceived social support. The 7-point Likert scale scores range from 12 to 84. Those who score 12 to 60 experience low to medium levels of perceived social support. However, those scoring 60 and above have a high level of perceived social support [21].

### Analysis

We used the Statistical Package for Social Sciences (SPSSTM) software Version 23 for analysis. Firstly, we described the continuous variables in frequencies, percentages, and mean ± standard deviation (Sd). Then, normality testing using the Kolmogorov test and Shapiro–Wilk test were employed to assess the distribution of the dependent variable (GAD-7 scores-continuous variable). Secondly, we used the Chi-square tests to assess the association between the dependent variable (anxiety) and independent variables. Lastly, a multivariable logistic regression model was employed. We computed the association’s effect size in adjusted odds ratios with a 95% CI and a P-value of 0.05 (two-tailed).

## Results

### Sample realization

**Fig. 1:**
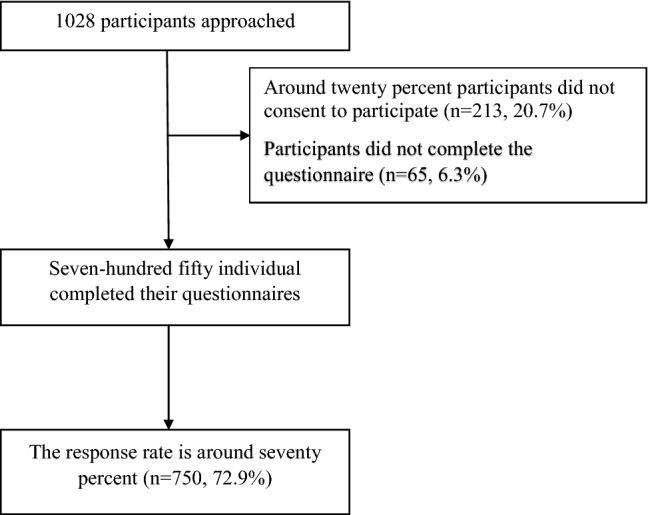
Flow chart of participants (N = 750)

From the total of 1,028 participants, 750 individuals completed their questionnaires, resulting in a response rate of approximately 70% (n = 750, 72.9%). Around 20% (n = 213, 20.7%) of the participants did not consent to participate and n = 65, 6.3% participants did not complete the questionnaire as seen in Fig. 1.

### Demographics and clinical profile of sample

The mean age of adolescents was 16 ± 0.9; the mode was 16 years. Females constituted the predominant gender (n = 537, 71.6%). Regarding the educational level, the third-year students reported the highest participation (n = 457, 60.9%). Furthermore, the non-Qatari participation was higher (n = 463, 61.7%) than the Qatari participation.

### Anxiety prevalence

The reported GAD-7 scores ranged between 0 and 21 with a mean score of 8.17, mode 4, and median 7. The curve showed skewness 0.5 (symmetrical) and kurtosis -0.83 (positive light tail). The Kolmogorov test results were Kolmogorov value = 0.11, p = 0.0001, and the results of the Shapiro–Wilk were Shapiro value = 0.93, p = 0.0001.

**Fig. 2:**
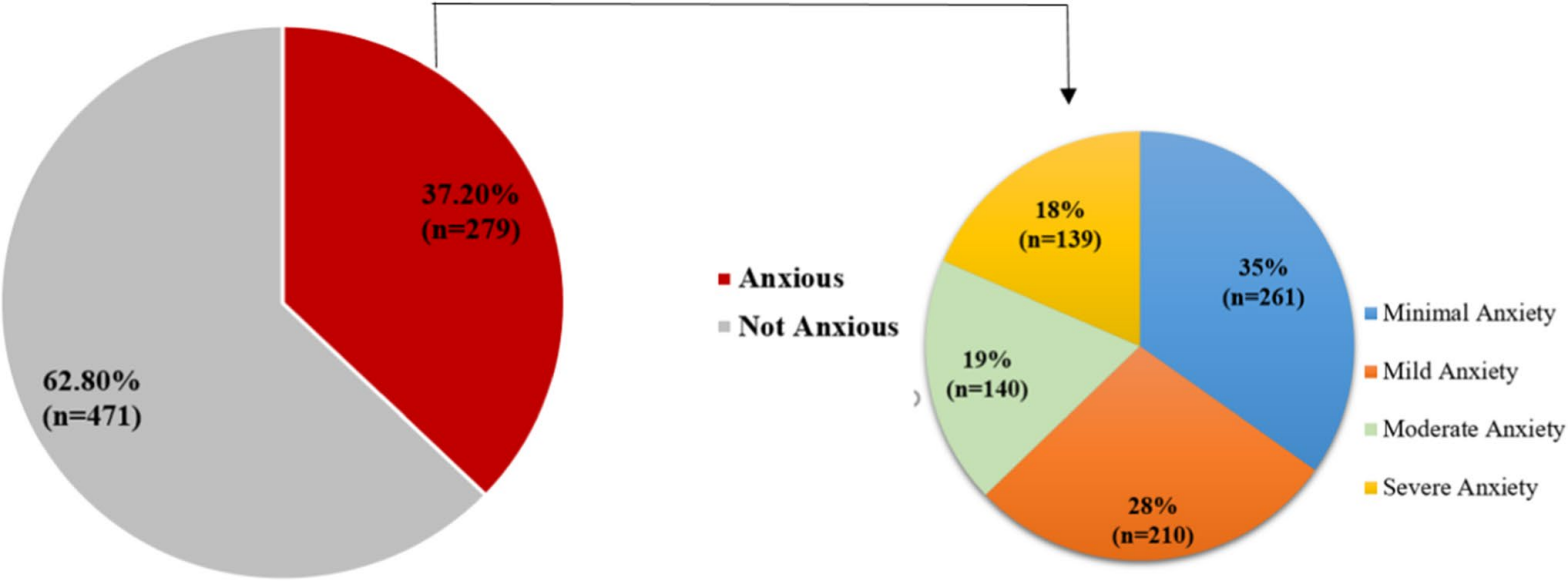
Distribution of anxiety among the participant’s attending independent secondary schools in Qatar, 2021 (N = 750)

Around one-fourth of the participants (n = 279, 37.2%) reported anxiety symptoms (total GAD score ≥ 10). The severity of anxiety symptoms showed minimal anxiety (n = 261, 34.8%), mild anxiety (n = 210, 28%), moderate anxiety (n = 140, 18.7%) and severe anxiety (n = 139, 18.5%) as seen in Fig. 2.

### Determinants

Female gender and participants with comorbidities were highly associated with anxiety. Nationality and other factors were not statistically associated with anxiety, as seen in Table 1.

**Table 1:** The socio-demographic and health-related variables association with anxiety among participants attending independent secondary schools in Qatar (N = 750)

	Anxiety (GAD-7 ≥ 10)							
	Anxious		Not Anxious					
	n	%	n	%	*χ*^2^	OR	95% [ CI]	p-value
I-Sociodemographic								
Gender								
Male Female	60 219	(28.2) (40.8)	153 318	(71.8) (59.2)	10.3	1.7	[1.2–2.4]	0.001*
Nationality								
Qatari Non-Qatari	112 167	(39.0) (36.1)	175 296	(61.0) (63.9)	0.66	0.8	[0.6–1.1]	0.416
II-Health Factors								
Did you get anxious during COVID-19 pandemic?								
Yes No	238 41	(48.2) (16.0)	256 215	(51.8) (84.0)	74.6	4.8	[3.3–7.1]	0.0001*
Did anyone in your family tested positive to COVID-19?								
Yes No	112 167	(38.9) (36.1)	176 295	(61.1) (63.9)	0.5	1.1	[0.8–1.5]	0.451
Are you under medical follow-up due to any of the following comorbidities?								
Yes No	40 239	(58.0) (35.1)	29 442	(42.0) (64.9)	14.0	2.5	[1.5–4.2]	0.0001*

*GAD-7* General anxiety Disorder-7, *OR* Odd Ratio, *CI* Confidence Interval

*p-value ≤ 0.05

Regarding the participants’ behavior, parenting style, and utilization of electronics for more than 12 h per day were statistically associated with anxiety, as seen in Table 2.

**Table 2:** The behavioral-related variables association with anxiety among participants attending independent secondary schools in Qatar (N = 750)

	Anxiety (GAD-7 ≥ 10)							
	Anxious		Not Anxious					
	n	%	n	%	*χ*^2^	OR	95% [CI]	p-value
Parenting style index								
Authoritative Permissive	111 168	(44.2) (33.7)	140 331	(55.8) (66.3)	7.9	1.5	[1.1–2.1]	0.005*
Increase in electronic consumption								
Yes No	250 29	(37.5) (34.5)	416 55	(62.5) (65.6)	0.29	1.1	[0.7–1.8]	0.59
Hours of utilizing electronics per day								
< 12 h > 12 h	197 82	(34.9) (44.3)	368 103	(65.1) (55.7)	5.33	1.4	[1.1–2.0]	0.021*
Perceived Social Support								
Low–medium PSS High PSS	146 133	(51.4) (28.5)	138 333	(48.6) (71.5)	39.4	2.5	[1.8–3.4]	0.0001*

*GAD-7* General anxiety Disorder-7, *OR* Odd Ratio, *CI* Confidence Interval

*p-value ≤ 0.05

Participants subjected to isolation or quarantine were more anxious than those not exposed to these COVID-19 management strategies. Those who used a mask, practiced social distancing, and hand hygiene protocol were inversely associated with anxiety. Furthermore, the participants who attended gatherings or crowded areas were typically more anxious, as seen in Table 3.

**Table 3:** COVID-19 related management association with anxiety among participants attending independent secondary schools in Qatar (N = 750)

COVID-19 related management questions	Anxiety (GAD-7 ≥ 10)							
	Anxious		Not Anxious					
	n	(%)	n	(%)	*χ*^2^	OR	95% [CI]	p-value
Did the time you use on social media platform increase during the COVID pandemic								
Yes No	250 29	(37.5) (34.5)	416 55	(62.5) (65.5)	0.29	1.1	[0.7–1.2]	0.59
Do you browse through social media platforms such as Facebook, twitter, Instagram, snapchat, Tik-Tok?								
Never used these website and Internet	11	(32.4)	23	(67.6)	8.3	–-	–-	0.039*
Not using websites but using internet	19	(40.4)	28	(59.6)				
I am using it sometimes	45	(28.0)	116	(72.0)				
I am using it daily	204	(40.0)	471	(62.8)				
Did you test positive for COVID-19?								
Yes No	49 230	(39.8) (36.7)	74 397	(60.2) (63.3)	0.4	1.1	[0.7–1.6]	0.508
Did any of your family members test positive for COVID- 19?								
Yes No	112 167	(38.9) (36.1)	176 296	(61.1) (63.9)	0.57	1.1	[0.8–1.5]	0.451
Were you quarantined due to COVID-19?								
Yes No	185 94	(41.3) (31.1)	263 208	(58.7) (68.9)	7.9	1.5	[1.1–2.1]	0.005*
Were you isolated due to COVID-19?								
Yes No	154 125	(44.9) (30.7)	189 282	(55.1) (69.3)	16.0	1.8	[1.3–2.4]	0.0001*
Do you wear face mask in public places (shopping malls, Supermarket, school)?								
Yes No	272 7	(37.0) (50.0)	464 7	(63.0) (50.0)	1.001	0.5	[0.2–1.6]	0.317
Do you wear face mask in social gatherings with family, friends, invites or to majlis?								
Yes No	93 186	(30.6) (41.7)	211 260	(69.4) (58.3)	9.5	0.6	[0.4–0.8]	0.002*
Do you have any face mask ready for use when you leave home?								
Yes No	260 19	(37.4) (35.2)	436 35	(62.6) (64.8)	0.1	1.0	[0.6–1.9]	0.750
Have you attended lately any social gathering with large number of people?								
Yes No	76 203	(49.4) (34.1)	78 393	(50.6) (65.9)	12.2	1.8	[1.3–2.7]	0.0001*
Have you attended lately any crowded places?								
Yes No	110 169	(44.0) (33.8)	140 331	(56.0) (66.2)	7.4	1.5	[1.1–2.1]	0.006*
Do you practice social distancing?								
Yes No	201 78	(33.4) (52.7)	401 70	(66.6) (47.3)	18.9	0.4	[0.3–0.6]	0.0001*
Have you lately washed your hands frequently using water and soap for 20 s, especially after; going to crowded areas, blowing nose, coughing and sneezing?								
Yes No	188 91	(34.4) (44.6)	358 113	(65.6) (55.4)	6.5	0.6	[0.4–0.9]	0.010*

*GAD-7*  General anxiety Disorder-7, *OR*  Odd Ratio, *CI* Confidence Interval

* p-value < 0.05

#### Predictors

The critical factors that were determined to be significant in the final model of the multi-logistic regression included those students exposed to COVID-19 isolation, low to moderate perceived social support levels, and permissive parental style; these participants were more typically anxious. However, other factors such as female gender failed to predict anxiety, as seen in Table 4.

**Table 4:** Logistic regression model of the predictors of anxiety among participants attending independent secondary schools in Qatar (N = 750)

Explanatory variables	AOR	[95% CI of Exp (B)]	P value
Gender			
Male	1		
Female	1.45	[0.98–2.14]	0.0612
Did you get anxious during COVID-19 pandemic?			
No Yes	1 4.43	[2.97–6.62]	0.0001*
Parenting style Index			
Authoritative Permissive	1 1.74	[1.22–2.47]	0.0023*
Do you wear face mask in social gatherings with family, friends, invites or to the Majlis?			
No Yes	1 1.25	[0.87–1.79]	0.2282
Where have you quarantined due to COVID-19?			
No Yes	1 1.60	[0.7–3.2]	0.1881
Where you isolated due to COVID-19?			
No Yes	1 1.89	[1.27–2.80]	0.0013*
Have you attended lately any crowded places?			
Yes No	1 0.89	[0.58–1.37]	0.6132
Have you attended lately any social gathering with large number of people?			
Yes No	1 0.90	[0.54–1.49]	0.7011
Do you practice social distancing?			
No Yes	1 2.04	[1.29–3.22]	0.0021*
Perceived Social Support			
High Low to moderate	1 2.46	[1.75–3.45]	0.0001*

*GAD-7* Generalized Anxiety Disorder 7 items, *AOR* adjusted Odd Ratio

*p-value ≤ 0.05; 1 = reference group

### Reliability of scales

The computed reliability of the utilized scales is based on Cronbach’s alpha of 0.92 for the multidimensional perceived social support scale, and 0.91 for the GAD-7 scale.

## Discussion

Anxiety was reported in 37.2% (n = 279) of the secondary school students in Qatar. Relatively, our data was lower than the results published in China, where the reported anxiety prevalence was 45.1% [22]. However, the results of our study show a higher prevalence than the expected anxiety level among adolescents in the GCC prior to the pandemic [4]. Furthermore, our prevalence was higher than the results reported in Saudi Arabia, Latin America, and Jordan among adolescents during the pandemic, which reported around 28% anxiety prevalence utilizing screening tools [23–25].

### Determinants of anxiety

Females were more commonly anxious than males. Similar to the Saudi Arabia results, female participants had 5.3% higher anxiety levels than male participants during the COVID-19 pandemic [23].

Our hypothesis that all COVID-19 management strategies increased anxiety levels among adolescents proved to be false. Surprisingly, adolescents who wore a face mask were less anxious than those who did not (OR 0.06;95% CI [0.4–0.8]). These findings were in line with another study conducted in China that indicated that wearing a face mask had a protective association with anxiety. In addition, students who rarely wore face masks had significantly higher odds of self-reported psychological distress (OR 2.59; 95% CI [2.41–2.79]) compared with students who always wore face masks [26].

On the other hand, physical isolation that was forced through the COVID-19 management strategies increased anxiety levels. Specifically, isolated adolescents were significantly more anxious (44.9%) than those who were not isolated (30.7%). However, lower anxiety prevalence was reported in a study conducted in California, in which 30% of adolescents isolated during the pandemic reported anxiety [27]. The differences in the reported prevalence between our study and California could be justified by the differences in the utilized measurement tools, as they used interviews to assess the anxiety disorders, while we used self-reported screening tools.

We explored the relationship between anxiety and parenting styles and found that an authoritative parenting style was highly associated with anxiety among adolescents (44.2%) compared to a permissive parenting style (33.7%). However, other studies showed that both parenting styles increased anxiety among adolescents. The study conflicted with our results, as we found that authoritative style was linked to higher anxiety [28].

Comparing our results to a systematic review on parenting style showed considerable heterogeneity in the different parenting styles, making it difficult to draw firm conclusions. This heterogeneity is seen since the studies used approximately 40 different instruments. In addition, there was notable variation in the parenting styles, even within studies that used the same measurement instruments [29].

Our data revealed that adolescents who attended crowded places were more anxious (44.0%) than those who did not attend crowd area (33.8%). Furthermore, those who attended social gatherings with many people were more anxious (49.4%) than those who did not attend social gatherings (34.1%). This is similar to the systematic review that revealed a high correlation between social gatherings and crowdedness and higher anxiety symptoms [30].

In regard to the level of perception of social support, we found that those who perceived low to medium social support (51.4%) were more anxious than those perceiving higher social support (28.5%). Similar patterns were reported in another study conducted among Chinese adolescent during the COVID-19 pandemic [31].

### Strengths and limitations

This is the first study in Qatar to investigate anxiety symptoms among adolescents during the COVID-19 pandemic. First, we utilized a highly sensitive cut-off point of a valid screening tool (GAD-7) to avoid misclassification bias. Additionally, all the measurement tools were tested for their face, content, and translation validity prior to data collection. Second, the setting was selected from independent secondary schools in Qatar, where healthy adolescents of all socioeconomic backgrounds allowed us to generalize results to the state of Qatar. Data collection process was anonymous through online surveys, which enabled students to overcome mental health stigmas and talk about their fears without judgment.

This study does have some limitations. First, we utilized non-probability convenient sampling that could limit the generalization of results. Second, the cross-sectional nature compromised causality and temporality between dependent outcome (anxiety) and independent variables.

## Conclusions

Anxiety among adolescents is common during a pandemic, which leads us to recommend screening for anxiety in a school setting. Furthermore, identifying adolescents with permissive parenting style, low to moderate perceived social support, adherence to social distancing and isolation due to COVID-19 pandemic may prove fruitful, as these students are at a higher risk of anxiety.

## Data Availability

The datasets used and/or analysed during the current study are available from the corresponding author on reasonable request.

## Abbreviations

COVID-19: Coronavirus Disease-2019
GAD: Generalized Anxiety Disorder
WHO: World Health Organization
GCC: Gulf Cooperation Council (GCC)
MSPSS: Multidimensional Scale of Perceived Social Support
